# Drought responses of three closely related *Caragana* species: implication for their vicarious distribution

**DOI:** 10.1002/ece3.2044

**Published:** 2016-03-18

**Authors:** Fei Ma, Xiaofan Na, Tingting Xu

**Affiliations:** ^1^ New Technology Application, Research and Development CenterNingxia UniversityYinchuan750021China; ^2^ School of Life ScienceNingxia UniversityYinchuan750021China

**Keywords:** *Caragana*, drought stress, growth, vicarious distribution, water use efficiency

## Abstract

Drought is a major environmental constraint affecting growth and distribution of plants in the desert region of the Inner Mongolia plateau. *Caragana microphylla, C. liouana,* and *C. korshinskii* are phylogenetically close but distribute vicariously in Mongolia plateau. To gain a better understanding of the ecological differentiation between these three species, we examined the leaf gas exchange, growth, water use efficiency, biomass accumulation and allocation by subjecting their seedlings to low and high drought treatments in a glasshouse. Increasing drought stress had a significant effect on many aspects of seedling performance in all species, but the physiology and growth varied with species in response to drought. *C. korshinskii* exhibited lower sensitivity of photosynthetic rate and growth, lower specific leaf area, higher biomass allocation to roots, higher levels of water use efficiency to drought compared with the other two species. Only minor interspecific differences in growth performances were observed between *C. liouana* and *C. microphylla*. These results indicated that faster seedling growth rate and more efficient water use of *C. korshinskii* should confer increased drought tolerance and facilitate its establishment in more severe drought regions relative to *C. liouana* and *C. microphylla*.

## Introduction

Vicarious distribution in closely related species is common in plants, which is defined as one species replacing another in an ecosystem and becoming a geographical replacement (Zhang 2000; Zhao 2002). However, ecophysiological basis for evolutionary differentiation of plant species that display vicarious distribution remains poorly understood. As various abiotic and biotic factors play key roles in plant growth and distribution, examining the responses of plants species to major ecological gradients (such as temperature and water availability) is thus an important step toward understanding their ecological differentiation.

The genus *Caragana*, belongs to the family *Leguminosae*, contains more than 80 species worldwide and often dominantly occupies cold‐temperate dry and arid scrublands, montane meadows, and deserts (Zhang et al. 2002). In China, *Caragana* species are well known for their role in sand fixation, and as fodder, green manure, fuel, honey resource, as well as medical applications (Ma et al. 2008). Due to its environmental benefit and economic value, this genus has been attracting increasing attention (Zhou et al. 2005; Meng et al. 2009; Zhang et al. 2009; Niu et al. 2013). *Caragana microphylla, C. liouana,* and *C. korshinskii* are three of the most common species in the desert region of the Inner Mongolia plateau. The three species are phylogenetic close and derived from the same ancient species, but now *C. microphylla* mainly occurs in the eastern plateau, *C. liouana* in middle, and *C. korshinskii* in the west of the plateau (Zhang et al. 2009). As such, these three species provide an ideal system to study physiological mechanisms underlying their ecological differentiation, and the interference with phylogeny and life history among species can be avoided (Liu et al. 2012). Recently, a morphological and physiological comparison among the three species in different areas of China has been described (Ma et al. 2003a, 2003b). And it has been found that the formation of interspecific alternative distribution was a result of plant adaptation to its natural environments and closely correlated with species morphological and physiological characteristics. However, a comprehensive understanding of how these three species respond to changes in soil water availability is still lacking.

In arid and semiarid environments, the maintenance of a positive carbon balance under drought stress is key to drought tolerance in plants. Leaf physiological traits such as photosynthetic capacity, stomatal conductance, water use efficiency, as well as the stable carbon and nitrogen isotope ratios (*δ* ^13^C and *δ* ^15^N) which are indicators for integrated water use efficiency and nitrogen fixation, have significant influence on net carbon gain and plant growth (Farquhar et al. 1989; Wright et al. 2004; Schulze et al. 2014). The relative growth rate (RGR), together with its components including net assimilation rate (NAR), leaf area ratio (LAR), specific leaf area (SLA), and leaf mass fraction (LMF), is a measure of plant growth efficiency (Shipley 2000; Elberse et al. 2003). In the present study, we subjected seedling of these three species to two water treatments (low and high drought treatments) and examined the traits mentioned above. The aim of this experiment was to test differences in drought tolerance among the three species and the corresponding distribution area of each species.

## Materials and Methods

The experiment was conducted at the University of Ningxia, Yinchuan, China (39°17′N, 108°02′E). Seeds of *Caragana microphylla*,* C. liouana,* and *C. korshinskii* were collected from trees growing in three different regional populations. Seeds of *C. microphylla* were collected from Ulan'aodu in the Khorchin Sandland, Inner Mongolia, and those of *C. liouana* and *C. korshinskii* were collected from Yanchi in the Mu Us Sandland, Ningxia Hui Autonomous Region, and Minqin in the Tengger Desert, Gansu Province. The corresponding mean annual rainfall (MAR) values for Ulan'aodu, Yanchi, and Minqin are 311, 297, and 110 mm, while the mean annual temperatures (MAT) are 6.1, 8.1, and 8.8°C, respectively.

Seeds were germinated and grown in growth chambers for 1 month. A total of 54 seedlings of each species with no statistical differences in height and size were selected and replanted into plastic pots with a homogeneous mixture (sand and perlite, 1:1 by volume) (three seedlings per pot). All pots were randomly placed in a canopied and naturally lit glasshouse with sides being always opened for aeration throughout the experiment to maintain the ambient outside temperature. Prior to the experiment, all pots were periodically watered to maximal field capacity (FC) for 2 months to allow the seedlings to become established.

The study was carried out with 3‐month‐old seedlings from July through September in 2014, lasting for 60 days. For each species, 14 pots were selected and divided into two lots of seven pots (low and high drought stress treatments). The remaining pots were used to determine the initial biomass. The low and high drought stress treatments were achieved by watering to 80% and 30% of FC. Soil water content was maintained by weighing the pots every 2 days and then immediately rewatering to the designated water level. The soil water content was maintained at 22–24% and 9–10% for low and high drought treatment, respectively. A total of 8 g slow release fertilizer was added before the experiment.

### Leaf gas exchange

Instantaneous gas exchange variables including net photosynthetic rate (*P*_N_), stomatal conductance (*g*_s_), transpiration rate (*E*), and intercellular CO_2_ concentration ratio (*C*_i_) were measured under artificial, saturating photon flux density (1500 *μ*mol·m^−2^·s^−1^) at an ambient CO_2_ concentration of 380 *μ*mol·mol^−1^ using a LI‐6400XT infrared gas analyzer (IRGA; LI‐COR, Lincoln, NE). Measurements were taken between 09: 00 and 11: 30 h on a sunny day (August 7, 2014) from five plants in each treatment for each species. During measurements, leaf temperature and leaf‐to‐air vapor pressure deficit were maintained at 31.79 ± 0.12°C and 1.94 ± 0.03 Kpa, respectively. Instantaneous water use efficiency (WUE_i_) was calculated as *P*_N_/*E*. Stomatal limitation value (*L*_s_) was defined as 1 − *C*_i_/*C*_a_.

### Growth

Due to possible within‐pot effects, such as competition for resources, each pot was considered to be a single replicate. To estimate the biomass production during the experiment, four pots (12 seedlings) from each species were harvested at the beginning of the experiment (t1) and five pots (15 seedlings) were harvested at the end of the experiment (t2). From each pot, the three seedlings were combined and then divided into three parts: leaves, stems, and roots. Biomass was dried for 48 h at 80°C in an oven and weights were divided by three to determine per plant values from the per pot values. Total biomass (per plant) is reported on a dry weight basis. Different biomass partition parameters like root shoot ratio (RSR), root mass fraction (RMF), stem mass fraction (SMF), LMF, LAR, and SLA were determined. Based on these data, RGR and NAR for each species and each treatment were also computed (Nagakura et al. 2004).

### Chemical and isotope analysis

Leaves with a dry weight of *c*. 0.2 g were collected from each seedling on which photosynthetic rate was made and then finely ground with a TissueLyser (Retsch, Haan, Germany). The leaf samples were then analyzed for *δ* ^13^C and *δ* ^15^N by isotope mass spectrometer (Finnigan Delta Plus, Bremen, Germany). Carbon and nitrogen isotope values were expressed relative to the Pee Dee Belemnite standard as the ratio (‰): *δZ* = (*R*_sample_/*R*_standard_ − 1) × 1000, where *Z* is the heavy isotope of either nitrogen or carbon, and *R* is the ratios of ^13^C/^12^C or ^15^N/^14^N in the sample and the standard. Carbon and nitrogen contents (LCC and LNC) in leaves were also determined.

### Statistical analysis

The effects of the drought treatments, the tested species, and their interactions on the examined variables were determined by general linear model (Proc GLM) using a SPSS software package (SPSS Inc., Chicago, IL). A Tukey's honestly significant difference test was used for multiple comparisons of means. Linear regressions between variables were performed using SigmaPlot version 12.5 (Systat Software, Inc., Chicago, IL).

## Results

### Leaf gas exchange

**Figure 1:**
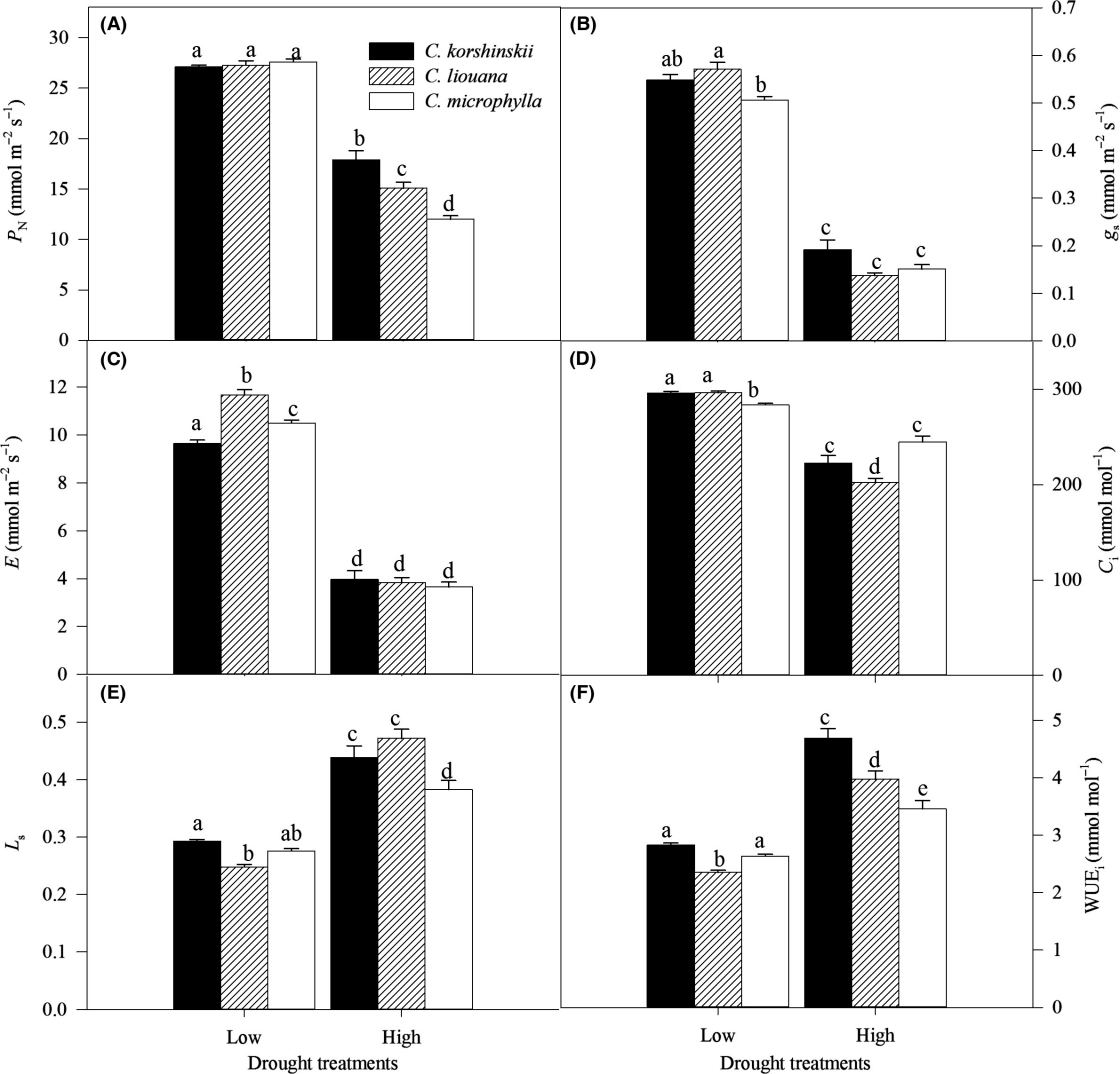
Net photosynthetic rate (*P*_N_, A), stomatal conductance (*g*_s_, B), transpiration rate (*E*, C), intercellular CO_2_ concentration (*C*_i_, D), stomatal limitation value (*L*_s_, E), and water use efficiency (WUE_i_, F) of *Caragana korshinskii*,* C. liouana,* and *C. microphylla* subjected to low and high drought treatments. Different letters denote significant differences (*P *<* *0.05) between means for species and treatments.

**Figure 2:**
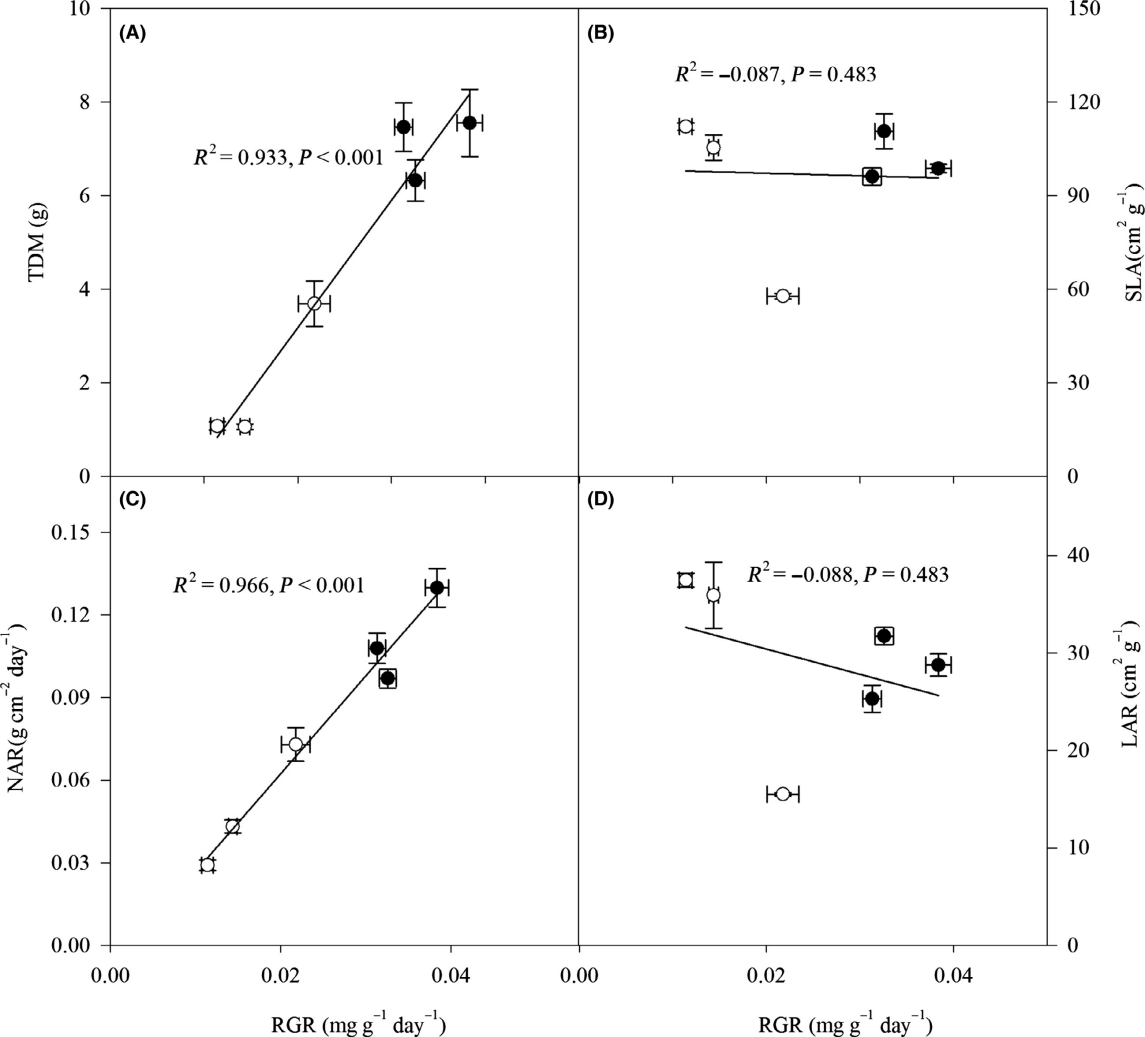
Relative growth rate (RGR) in relation to total dry mass (TDM, A), specific leaf area (SLA, B), net assimilation rate (NAR, C), and leaf area ratio (LAR, D) of *Caragana korshinskii*,* C. liouana,* and *C. microphylla* subjected to low (filled circles) and high (empty circles) drought treatments. The coefficient of determination (*R* ^2^) and significance are shown for each regression.

The leaf gas exchange parameters like *P*_N_, *g*_s_, *C*_i_, *E*,* L*_s_, and WUE_i_ were significantly affected by species, drought, and their interactions (Table 1). *P*_N_, *g*_s_, *E,* and *C*_i_ decreased significantly in all species with increasing drought stress, while *L*_s_ and WUE_i_ significantly increased (Fig. 1). However, the changed extent of *P*_N_ and WUE_i_ to drought was highest in *C. microphylla*, followed by *C. liouana*, and lowest in *C. korshinskii*. These results directly resulted in significantly higher *P*_N_ and WUE_i_ in *C. korshinskii* than those in *C. liouana* and *C. microphylla*. Additionally, there was a strong correlation between *P*_N_ and *g*_s_ across treatments in the study (Fig. 2).

**Table 1:** Comparison of all variables measured in the experiment. The *P*‐values are presented for the watering treatments, species, and their interactions

Variables	Abbrev.	Species (*S*)	Treatment (*T*)	*S* × *T*
Net photosynthetic rate	*P*_N_	19.03***	1156.20***	26.70***
Stomatal conductance	*g*_s_	4.91**	1073.96***	4.53*
Intercellular CO_2_ concentration	*C*_i_	6.71**	489.94***	27.50***
Transpiration rate	*E*	9.35***	1417.67***	10.88***
Stomatal limitation value	*L*_s_	9.14***	413.83***	20.25***
instantaneous water use efficiency	WUE_i_	38.63***	428.39***	22.42***
Total dry mass (g)	TDM	8.95**	195.93***	4.53*
Leaf dry mass (g)	LDM	7.59**	139.71***	6.89**
Stem dry mass (g)	SDM	3.69*	164.82***	2.20
Root dry mass (g)	RDM	9.41**	99.60***	5.97**
Height increment (cm)	*H*_t_	9.78***	167.55***	2.31
Stem diameter increment (mm)	*S*_d_	1.45	46.90***	7.39**
Total leaf area (cm^2^)	TLA	0.39	324.08***	1.05
Specific leaf area (cm^2^·g^−1^)	SLA	64.14***	15.31***	30.65***
Leaf area ratio (cm^2^·g^−1^)	LAR	43.38***	0.61	16.45***
Leaf mass ratio (g·g^−1^)	LMR	2.85*	11.63**	2.48
Stem mass ratio (g·g^−1^)	SMR	0.78	11.66**	0.20
Root mass ratio (g·g^−1^)	RMR	0.15	82.98***	0.90
Root shoot ratio (g·g^−1^)	RSR	2.85	3.05	1.87
Relative growth rate (mg·g^−1^·day^−1^)	RGR	10.84***	405.98***	23.94***
Net assimilation rate (g·cm^−2^·day^−1^)	NAR	64.14***	262.69***	15.06***
Carbon isotope ratio (‰)	*δ* ^13^C	10.33**	307.42***	2.90
Nitrogen isotope ratio (‰)	*δ* ^15^N	168.07***	4080.15***	165.53***
Carbon nitrogen ratio	C/N	365.11***	7.59*	32.71***

*P < 0.05; **P < 0.01; ***P < 0.001.

### Biomass and leaf area

The effects of drought, species, and their interactions on TDM, LDM, RDM, SLA, SDM, TLA, and LAR were visible (Table 1). Under drought conditions, the TDM, LDM, SDM, and RDM were significantly higher in *C. korshinskii* than those in *C. liouana* and *C. microphylla*, but the latter two species showed no significant differences (Table 2). Although TLA decreased in all species with increasing drought stress, the differences between species were not significant. Only SLA and LAR of *C. korshinskii* significantly decreased from low to high drought stress, resulting in lower values of SLA and LAR in comparison with those of the other two species. In addition, regardless of the drought treatments and species, a strong and positive relationship between *P*_N_ and TDM we observed (Fig. 2).

**Table 2:** Growth variables of three *Caragana* species subjected to control and drought treatments

Variables	Abbrev.	*C. korshinskii*		*C. liouana*		*C. microphylla*	
		Control	Drought	Control	Drought	Control	Drought
Total dry mass (g)	TDM	7.46 ± 0.52 a	3.69 ± 0.48 b	6.32 ± 0.44 a	1.07 ± 0.09 c	7.55 ± 0.71 a	1.06 ± 0.05 c
Leave dry mass (g)	LDM	1.77 ± 0.09 a	0.88 ± 0.12 b	1.38 ± 0.20 c	0.34 ± 0.11 d	2.16 ± 0.14 e	0.37 ± 0.05 d
Stem dry mass (g)	SDM	2.05 ± 0.23 a	0.61 ± 0.14 b	1.60 ± 0.04 c	0.32 ± 0.11 bd	1.96 ± 0.20 ac	0.09 ± 0.02 d
Root dry mass (g)	RDM	3.36 ± 0.28 a	2.20 ± 0.28 b	2.74 ± 0.12 ab	0.82 ± 0.14 c	3.42 ± 0.40 a	0.60 ± 0.06 c
Height increase (cm)	*H*_t_	39.58 ± 1.98 a	22.27 ± 1.19 b	35.78 ± 1.21 a	14.39 ± 0.91 c	35.01 ± 3.52 a	8.77 ± 1.25 c
Stem diameter increase (mm)	*S*_d_	2.08 ± 0.16 a	1.87 ± 0.15 a	2.85 ± 0.10 b	1.64 ± 0.14 a	3.00 ± 0.28 b	1.46 ± 0.11 a
Total leaf area (cm^2^)	TLA	219.81 ± 4.06 a	78.26 ± 8.10 b	235.10 ± 20.67 a	81.28 ± 2.20 b	237.91 ± 11.31 a	65.84 ± 5.62 b
Specific leaf area (cm^2^·g^−1^)	SLA	96.11 ± 2.79 a	57.82 ± 0.88 b	110.62 ± 5.58 c	112.12 ± 13 c	98.72 ± 1.36 a	105.38 ± 4.04 ac
Leaf area ratio (cm^2^·g^−1^)	LAR	25.28 ± 1.38 a	15.51 ± 0.16 b	31.72 ± 0.91 cd	37.46 ± 0.72 e	28.74 ± 1.13 ac	35.91 ± 3.39 de
Leaf mass ratio (g·cm^−2^)	LMR	0.25 ± 0.01 a	0.24 ± 0.00 a	0.26 ± 0.02 a	0.19 ± 0.01 b	0.28 ± 0.01 a	0.25 ± 0.05 a
Stem mass ratio (g·g^−1^)	SMR	0.28 ± 0.02 a	0.16 ± 0.04 b	0.28 ± 0.02 a	0.19 ± 0.05 b	0.26 ± 0.01 ab	0.18 ± 0.07 b
Root mass ratio (g·g^−1^)	RMR	0.47 ± 0.00 a	0.60 ± 0.02 b	0.46 ± 0.01 a	0.62 ± 0.03 b	0.45 ± 0.02 a	0.57 ± 0.06 b
Root shoot ratio (g·g^−1^)	RSR	0.80 ± 0.01 a	1.08 ± 0.08 b	0.76 ± 0.04 a	0.76 ± 0.02 a	0.78 ± 0.04 a	0.91 ± 0.12 a
Relative growth rate (mg·g^−1^·day^−1^)	RGR	31.30 ± 0.98 a	21.76 ± 1.68 b	32.57 ± 0.10 a	11.38 ± 0.68 c	38.36 ± 1.36 d	14.35 ± 0.51 c
Net assimilation rate (g·cm^−2^·day^−1^)	NAR	0.108 ± 0.005 a	0.073 ± 0.006 b	0.097 ± 0.003 a	0.029 ± 0.002 c	0.130 ± 0.007 d	0.043 ± 0.002 c

Different letters denote significant differences (ANOVA test, *P *<* *0.05) between means for species within each irrigation treatment. Each point represents the mean ± SE.

### Biomass allocation

The drought treatment had a significant effect on LMF, SMF, and RMF, while the species effect was significant only in LMF and there was no interactive effect on LMF, SMF, RMF, and RSR (Table 1). From low to high drought stress, all species showed an increase in RMF and a decrease in SMF, and slight changes in LMF except for *C. liouana* whose LMF increased (Table 2). *C. korshinskii* tended to increase more biomass to roots, whereas there were no significant differences in RSR for *C. liouana* and *C. microphylla* between treatments (Table 2).

### Plant growth

**Figure 3:**
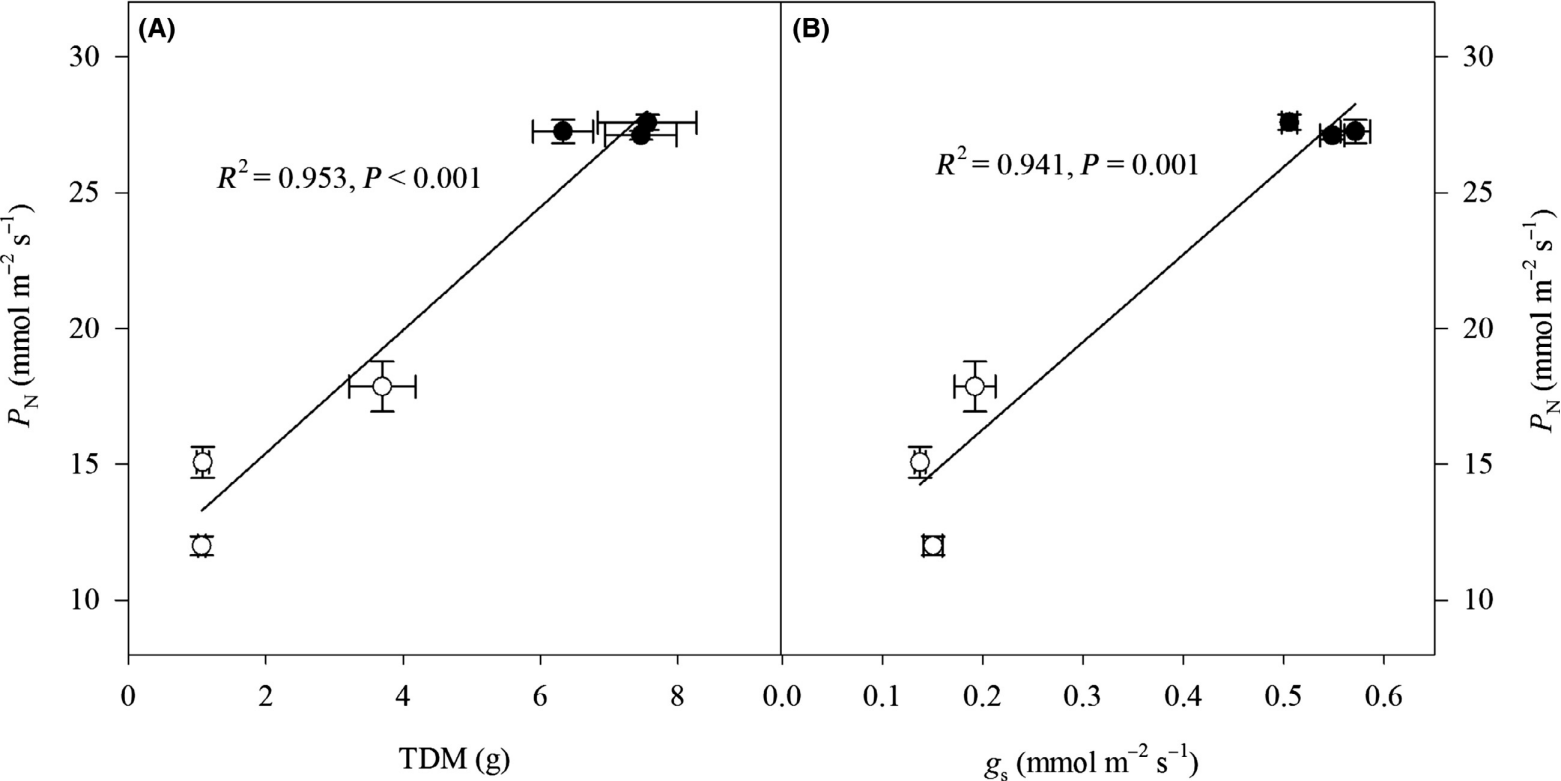
Net photosynthetic rate (*P*_N_) in relation to total dry mass (TDM) and stomatal conductance (*g*_s_) of *Caragana korshinskii*,* C. liouana,* and *C. microphylla* subjected to low (filled circles) and high (empty circles) drought treatments. The coefficient of determination (*R* ^2^) and significance are shown for each regression.

The effects of drought, species, and their interactions were significant on RGR, NAR, and height increment (*H*_t_), while the increase of stem diameter (*S*_d_) was significantly influenced by drought and the interactive effect (Table 1). Although increasing drought stress resulted in significant decreases in RGR, NAR, shoot, and stem growth in all species, *C. korshinskii* exhibited higher RGR NRA and *H*_t_ than *C. liouana* and *C. microphylla* (Table 2). In addition, regardless of the effect of drought and species, no significant relationship could be observed between RGR and LAR (*R* ^2^ = −0.088, *P* = 0.483), or SLA (*R* ^2^ = −0.087, *P* = 0.483) (Fig. 3). Hence, RGR was most likely a strict function of NAR, as indicated by the tight correlation between these two variables (*R* ^2^ = 0.966, *P* < 0.001) (Fig. 3).

### Leaf isotopes

**Figure 4:**
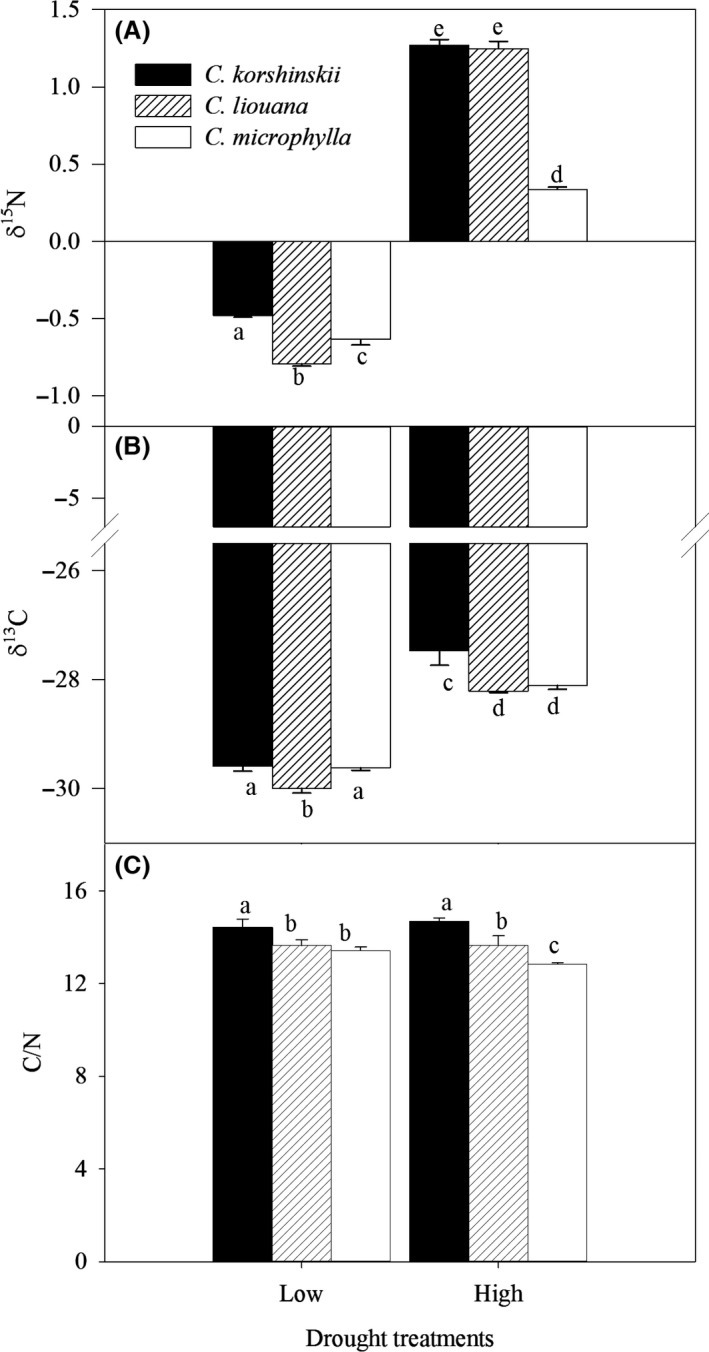
Nitrogen isotope ratios (A, *δ* ^15^N), carbon isotope ratios (B, *δ* ^13^C), and the ratios of carbon to nitrogen (C, C/N) of *Caragana korshinskii*,* C. liouana,* and *C. microphylla* subjected to low and high drought treatments. Different letters denote significant differences (*P* < 0.05) between means for species and treatments.

**Figure 5:**
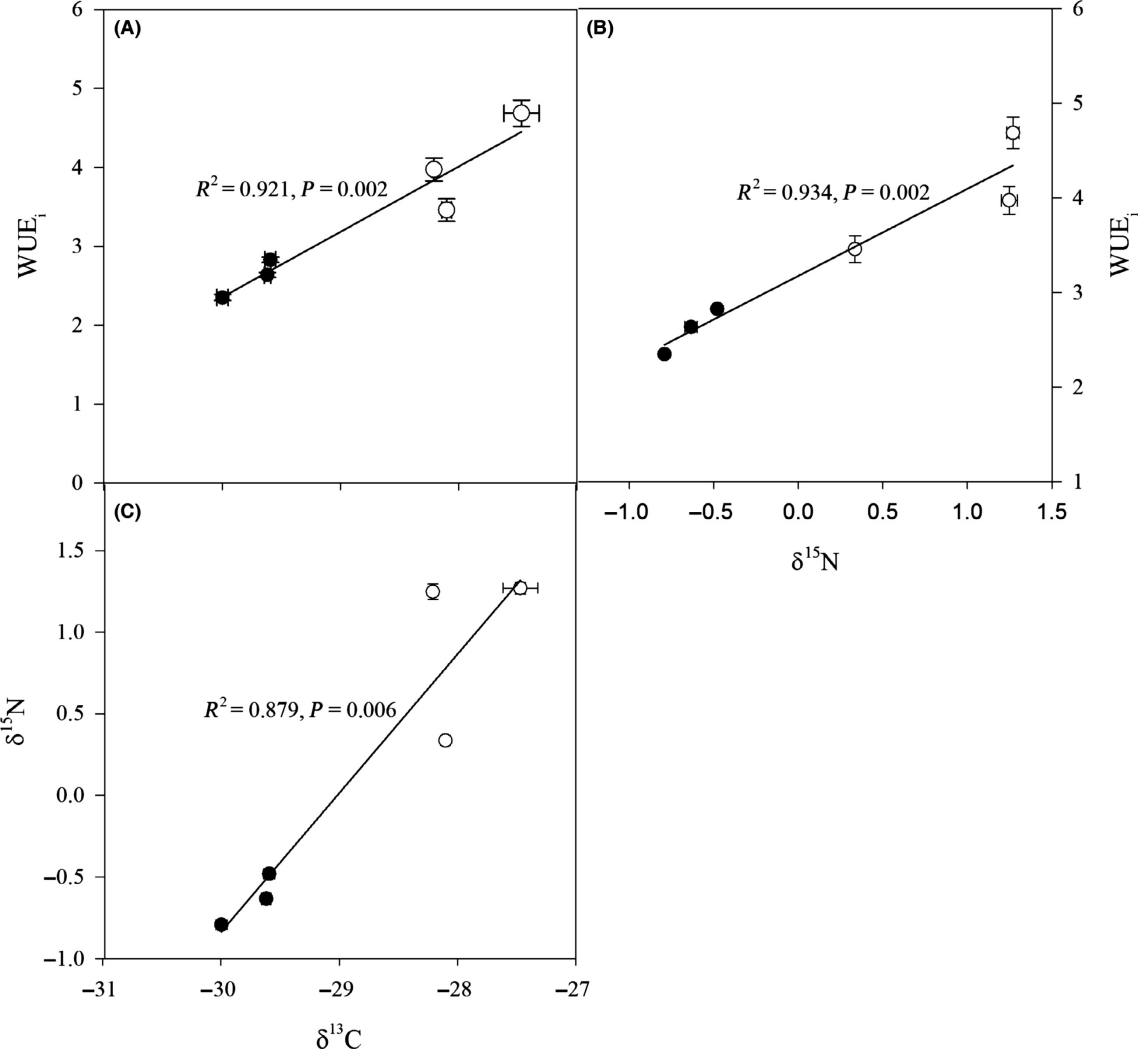
Relationships between the ratio of carbon and nitrogen isotopes (*δ* ^13^C and *δ* ^15^N) and water use efficiency (WUE_i_) of *Caragana korshinskii*,* C. liouana,* and *C. microphylla* subjected to low (filled circles) and high (empty circles) drought treatments. The coefficient of determination (*R* ^2^) and significance are shown for each regression.

Leaf carbon and nitrogen isotopes (*δ* ^13^C and *δ* ^15^N) were significantly affected by species and drought stress, but their interactive effect was only significant on *δ* ^15^N. With increasing drought stress, *δ* ^13^C and *δ* ^15^N increased in all species. However, *C. korshinskii* tended to exhibit significantly higher values of *δ* ^13^C than the other two species in high drought stress conditions while values of *δ* ^15^N were significantly higher in *C. korshinskii* and *C. liouana* than those in *C. microphylla* (Fig. 4). The ratio of carbon to nitrogen content (C/N) of the three species also showed a distinct response to drought, with *C. korshinskii* and *C. liouana* exhibiting slight changes in C/N between treatments but a decrease of C/N in *C. microphylla*. The C/N of *C. korshinskii* was also significantly higher than other two species at high drought treatment (Fig. 4). In addition, strong correlations between *δ* ^13^C and WUE_i_, as well as *δ* ^15^N, were observed (Fig. 5).

## Discussion

The three studied *Caragana* species are phylogenetically close but distribute vicariously in desert regions of the Inner Mongolia plateau. Drought is a major environmental constraint affecting growth and distribution of plants in these regions (Ma et al. 2004). Determining the interspecies differences in drought tolerance can aid in understanding their ecological differentiation.

Growth performance is essential for plant adaptation to drought (Richter et al. 2012). Plant species with higher drought tolerance exhibit less growth inhibition and have relatively higher growth and biomass production than drought‐sensitive species (Türkan et al. 2005; Couso and Fernández 2012). In this study, increasing drought stress decreased growth and biomass accumulation in all species, in conformity with other reports (Dias et al. 2007; Ma et al. 2010; Yang et al. 2014). We also found RGR was strongly correlated with TDM and NAR across treatments, but not with SLA (Fig. 2). This is also consistent with the observations of Poorter and Nagel (2000) that changes in NAR were mainly due to the decreases of RGR and, to a lesser extent, changes in SLA. However, the growth responses to increasing drought stress varied significantly with species, with the growth performance of *C. korshinskii* being less affected by high drought stress than that of *C. liouana* and *C. microphylla* (Table 2). Furthermore, at high drought stress treatment, *C. korshinskii* could exhibit significantly higher TDM, RGR, NAR, and *H*_t_ compared with the other two species. These results suggested *C. korshinskii* having a higher capacity to sustain growth and production in face of high drought stress conditions.

Drought affects plant growth by influencing leaf gas exchange rates (Zhang and Marshall 1994; Bacelar et al. 2007; Ma et al. 2010; Sapeta et al. 2013). In the present study, increasing drought stress restricted photosynthesis of all species but this negative effect was species dependent (Fig. 1). *C. korshinskii* exhibited lower sensitivity of *P*_N_ to increasing drought stress compared with the other two species and showed highest values of *P*_N_ under high drought conditions, which was followed orderly by *C. liouana* and *C. microphylla*. Moreover, there was a tight correlation between TDM and *P*_N_ across treatments, indicating that the primary contributing factor to higher growth rates and biomass accumulation of *C. korshinskii* was its leaf gas exchange rate.

Stomatal and nonstomatal limitations are considered to be the main causes of reduced photosynthesis in drought‐stressed plants (Flexas et al. 2014). In the present study, although *P*_N_ and *g*_s_ of the three species both decreased significantly with increasing drought stress, we found a significant and positive correlation between *P*_N_ and *g*_s_ across treatments (Fig. 3). We also found all species showed decreases of *C*_i_ and increases of *L*_s_ at high drought treatment. Form these results, we can therefore draw a conclusion that stomatal closure brought on by drought stress strongly accounted for the reduced photosynthesis (Michelozzi et al. 2011; Flexas et al. 2014).

Water use efficiency is essential for plants dealing with drought stress (Lambers et al. 1998) and plants with higher water use efficiency are generally able to survive drought stress (Jones 1993). In the present study, *P*_N_ and *E* of all species decreased significantly in response to drought stress, but the greater decreased extent of *E* compared to that of *P*_N_ produced an increased WUE_i_, which was agreed with our previous studies (Ma et al. 2010, 2014). Decreased *g*_s_ and *E* at high drought treatment would alternate the source of carbon fixation, leading to an increase of the ratios of ^13^C/^12^C and subsequent leaf carbon isotope ratio (*δ* ^13^C) (Fotelli et al. 2003; Kume et al. 2003). Our results also showed *δ* ^13^C was strongly increased by high drought stress in all species and the strong and positive correlation between *δ* ^13^C and WUE_i_ across treatments is also consistent with the results of previous studies (Farquhar et al. 1989; Osório and Pereira 1998; Zhang and Marshall 1994). However, water use efficiency (WUE_i_ and *δ* ^13^C) was significantly higher in *C. korshinskii* than those in *C. liouana* and *C. microphylla*, suggesting that *C. korshinskii* could use water more efficiently than the other two species, and subsequently contributing to its higher growth rate and biomass production. The lack of difference in growth performance and integrated water use efficiency (*δ* ^13^C) between *C. liouana* and *C. microphylla* may be due to the similar annual rainfall of regions of origin which influences plant response to drought (Mclean et al. 2014).

The higher growth and photosynthesis may also be related to the lower SLA and higher root shoot ratio. These two traits have been reported to provide fitness benefits for plants in arid environments (Ramírez‐Valiente et al. 2010; Yang et al. 2013). Low SLA allows the plant to avoid excess of water use, maintaining photosynthetic activity and carbon accumulation. Enhanced root shoot ratio allows the plants to absorb water and nutrients more efficiently. In the present study, a significant increase of RSR and a significant decrease of SLA were found in *C. korshinskii* while RSR and SLA of the other two species showed no significant change between treatments. These morphological changes of *C. korshinskii* therefore exhibited a more adaptive response to drought stress than the other two species (Yang et al. 2013).

The interpretation of leaf nitrogen isotope ratio (*δ* ^15^N) value is more complex than that of *δ* ^13^C because it integrates the conditions of nitrogen source, physiological mechanisms within the plant, and mycorrhizal associations (Michelsen et al. 1998; Kolb and Evans 2002). The significant enhancement of leaf *δ* ^15^N in all species under drought stress is similar to results of other studies (Austin and Sala 1999; Aranibar et al. 2004; Lotter et al. 2014; Schulze et al. 2014). One possible explanation is that more nitrogen is lost relative to turnover as the water availability decreases (Aranibar et al. 2004). As an N_2_‐fixing legume, *Caragana* species can form symbiotic relationship with arbuscular mycorrhizal fungi which provide N for the host but discriminate against ^15^N during fungal N assimilation (Hobbie et al. 2000). This symbiotic relationship is considered to very sensitive to environmental constraints, which is weakened under drought conditions (Arrese‐Igor et al. 2011). Unfortunately, our data do not allow us to discern which processes were occurring and at what intensity, but our data do show different N use strategies or microbe activities among species. Leaf *δ* ^15^N value has been related to water status due to the strong and positive correlation between *δ* ^15^N and *δ* ^13^C across treatments, as well as WUE_i_ (Stamatiadis et al. 2007).

In conclusion, interspecific differences were found in growth, gas exchange, water use efficiency, and biomass production among the three *Caragana* species subjected to increasing drought stress. *C. korshinskii* appeared to be the most drought‐tolerant species with superior growth performance and water use efficiency associated with lower SLA and higher root shoot ratio compared with the other two species. The growth and integrated water use efficiency of *C. microphylla* and *C. korshinskii* were not significantly different between treatments, which may be a result of similar MAR in their regions of origin. These results can certainly provide useful information in demonstrating the ecological differentiation among the three species. However, more population for each species and more interactive experiments between environmental factors, such as drought and heat stress, are needed in the future for deeper understanding the mechanisms underlying their vicariously distribution.

## Conflict of Interest

The authors declared no conflict of interests.
